# Fatty acid composition of multigenerational heat stressed adult Japanese quail (*Coturnix coturnix japonica)*

**DOI:** 10.1016/j.psj.2025.105916

**Published:** 2025-09-30

**Authors:** Linda Truong, Timothy J. Hackmann, Zhichao Zhang, Annie J. King

**Affiliations:** a Department of Animal Science, University of California Davis, Davis, CA 95616; b Department of Food Science, University of California Davis, Davis, CA 95616

**Keywords:** Selective breeding, Heat stress, Quail, Fatty acid, Tissue

## Abstract

As above average heat events are rising across the globe, it is imperative to understand the effects of heat stress on lipids of poultry species. The fatty acid composition of various organs of quail selectively bred for high fitness in heat stress has not been previously investigated. Therefore, this study treated 10 generations of quails with the following (1) random-bred in a thermoneutral temperature (22°C, **TN**), (2) random-bred in heat stress (31°C, **HS**), (3) selected for low feed conversion ratio (**FCR**) in heat stress and not exposed to heat stress (22°C, **TNS**), and (4) selected for low FCR in heat stress, then exposed to heat stress (31°C, **HSS**). Fatty acid composition was analyzed in feed, brain, liver, kidneys, and thighs of quail from the 10th generation. It was hypothesized that HS and HSS would have less polyunsaturated fatty acids (**PUFA**) in all organs due to oxidation and HS would have lower ratios of SFA:PUFA and n-6:n-3 compared to those that were not selectively bred. Data were analyzed using treatment, tissue, and their interaction as the main effects. Significance was determined at P ≤ 0.05. Results showed that of all organs analyzed, livers experienced the most variations in concentrations of fatty acids when compared by treatment. TN brains had less PUFA than that in both TNS and HSS. TN or TNS had more long chain PUFA and less saturated fatty acids than that in HS or HSS across all tissues. Thus, it can be concluded that selection for low FCR in heat stress may reduce oxidation of PUFA or increase retention of PUFA in the brain. However, overall, selectively breeding for low FCR in heat stress did not result in fatty acid differences compared to those that were random-bred in heat stress.

## Introduction

Heat stress is a top concern for global poultry producers (Hu et al., 2022). In heat stressed animals, it is energetically more efficient to deposit fat rather than protein; however, lipids are a class of nutrients that are particularly susceptible to heat stress induced oxidation (Noble and Cocchi, 1990; Renaudeau et al., 2012). During temperature stress, oxidative damage to the brain, heart, kidney, and liver, ultimately cause metabolic disruptions (Ren et al., 2018; Surai et al., 2016; Yang et al., 2024). While the effect of different poultry diets and heat stress on liver, brain, and muscle fatty acid composition have been studied, few have investigated how heat stress affects the fatty acid composition in brain or kidney in Japanese quail (Cherian, 2015; Tavaniello et al., 2017). Additionally, there is scarce literature on the use of selective breeding of poultry for low FCR in heat stress as a method to enhance tolerance to heat stress (Soleimani et al., 2011; Oluwagbenga and Fraley, 2023). Studies have shown that there are genetic and phenotypic differences in stress response between mice that have a difference of about 0.4 % in their genome (Keane et al., 2011; Terenina et al., 2019). Therefore, there is evidence that generations of selective breeding can affect stress response and subsequent metabolism. Selection for low FCR in heat stress can be economical to producers, result in healthier birds in heat stress, and provide better nutrients to the consumer.

Heat stress in poultry species cause a reduction in performance such as lower body weight and higher feed conversion ratio (Renaudeau et al., 2012; Slimen et al., 2016; Naga Raja Kumari and Narendra Nath, 2018; Liu et al., 2020; Papanikolaou et al., 2025). High fat retention in heat stressed poultry could be due to decreased peripheral lipolysis and decreased lipogenesis. Omega-3 fatty acids affect the expression of hepatic fatty acid synthase and can cause a decrease in triacylglyceride and adipose fat accumulation (Taouis et al., 2001). Therefore, liver fatty acids are important for overall poultry health. Poultry, such as quail meat and eggs, provide dietary essential fatty acids like n-6 and n-3 polyunsaturated fatty acids (**PUFA**) to people in various parts of the world (Alagawany et al., 2019). As poultry species are experiencing heat stress, there may be degradation of these essential PUFA; thus, affecting human health. The ideal human n-6:n-3 should be 1:1; however, current food consumption norms in economically developing countries where quail are consumed have a 20:1 n-6:n-3 (Alagawany et al., 2019). Therefore, investigation of liver fatty acids can help determine the fatty acids consumed by people.

The composition of dietary fat can influence the bird’s response to it during heat stress (Renaudeau et al., 2012). For example, n-3 PUFA were less resistant to oxidative damage than n-6 PUFA (Laviola and Macrì, 2013). In chicken, α-linolenic acid (**ALA**; C18:3 n-3) and linoleic acid (**LA**; C18:2 n-6) are dietary essential fatty acids because chickens lack the desaturase to insert a double bond beyond the −9 carbon (Cherian, 2015). With the current imbalance of n-6 to n-3 in current poultry diets, it was important to know if heat stress further changed their ratio. Of particular interest were arachidonic acid (**ARA**; C20:4 n-6) and docosahexaenoic acid (**DHA**; C22:6 n-3) due to their essentiality in many functions of the birds' body, particularly the brain and kidneys. Their essential functions include membrane structure, proper neural development, and visual acuity (Cherian, 2011, 2015). It has also been investigated that altering the fatty acid intake of hens can alter the fatty acid profile of chick brains (Whittle et al., 2024). Therefore, it is important to understand the effects of heat stress on the feed and subsequent adult tissues as it has an impact on future generations.

Avian kidneys are also highly susceptible to oxidative damage during temperature stress because they are one of the major organs for acid-base homeostasis (Farag and Alagawany, 2018; Ren et al., 2018). Approximately 50 to 70 % of the energy use in mammalian kidneys is dedicated to the sodium pump (Szabó et al., 2010). Also, it was shown that lipid composition, particularly DHA, in the kidney membrane can influence the activity of the sodium pump (Szabó et al., 2010). Due to the kidneys’ vital role for maintaining the acid-base balance during times of stress, understanding its fatty acid composition during heat stress in poultry is important (Sejian et al., 2015).

Therefore, this study aimed to determine if fatty acid composition of brain, liver, kidney, and thigh changed over generations of selection for low FCR in heat stressed Japanese quail. It is expected that, when compared to samples obtained from thermoneutral birds, there will be less PUFA in samples obtained from heat stressed quail due to oxidation and utilization in stress responses such as an increase in pro-inflammatory eicosanoids, which are derivatives of ARA. However, those selected for low FCR in heat stress may have more PUFAs than those that were not selected because of possible enhanced tolerance to heat stress. Decrease of PUFAs in the brain are expected because brain lipids are approximately 23.5 % PUFA and oxidation in the brain has been previously shown to be higher than other organs (Durairaj and Martin, 1971; Poureslami et al., 2010; Truong and King, 2023).

## Materials and methods

### Experimental design

Animal care and use was approved by the Institutional Animal Care and Use Committee at the University of California Davis (Protocols #19473 and 21370; Davis, CA; Approval dates 8/5/2016 and 8/20/2019, respectively).

**Fig. 1:**
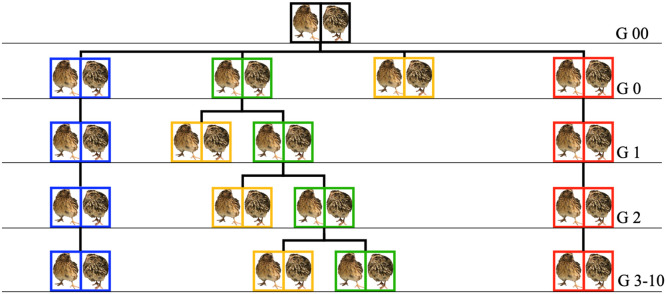
Diagram of breeding program. Black boxes in generation 00 represent the stock population, housed, and mated at 22°C. In generation 0, blue boxes represent the Thermoneutral (TN) treatment, housed and randomly mated at 22°C; green boxes represent the Thermoneutral sibling (TNS) treatment, housed and selectively bred at 22°C; yellow boxes represent the Heat-stressed siblings (HSS) treatment, housed at 31°C and not bred for the next generation; red boxes represent Heat-stressed (HS) treatment, housed and mated at 31°C. This breeding scheme repeated from generations 0 to 10.

Detailed description of selective breeding from generation 0 can be found in Fig. 1 and in Truong et al., 2023. The 10th generation was used in this study. Briefly, all birds were hatched at 33°C with 61 % RH. Wing bands were used to track lineage. They were then reared together in brooder cages until 3.5 weeks of age. At 3.5 weeks old, quail were sexed and separated into their respective treatments.

The 4 treatments were: (1) thermoneutral controls (22°C, **TN**), (2) thermoneutral siblings (22°C, **TNS**), (3) heat stress (31°C, **HS**), and (4) heat stressed siblings (31°C, **HSS**). HS was mated at 31°C. TNS was mated at 22°C and their offspring was evenly divided into 22°C (TNS) and 31°C (HSS). Only families with low feed conversion ratio (**FCR**) in HSS where mated. FCR was calculated using the following equation.

$$\text{FCR}=\left(\frac{\text{average}\;\text{feed}\;\text{intake}}{\text{average}\;\text{daily}\;\text{gain}\;}\right).$$

FCR was only compared to other families within treatment for HSS and HS. FCR was determined for 1 week starting at 31 d old for HSS and 38 d old for HS. ½ of males and ½ of females were classified as low FCR and the other ½ were classified as having high FCR. Heat exposure began 3 days after birds were acclimated to their adult cages. HS and HSS experienced cyclic heat stress in the following order.

1. 22 to 31°C between 0630 h to 1100 h (4.5 hours)
2. 31°C between 1100 h to 1630 h (5.5 hours)
3. 22°C between 1630 h to 2200 h (5.5 hours)
4. 22°C from 2200 h to 0630 h (8.5 hours).

HS and HSS experienced cyclic temperature fluctuations that would represent what poultry may experience if indoor temperature control was not possible. Therefore, the temperature of interest is the temperature at which heat stress occurred and not the mean temperature experienced by the quail. The relative humidity remained constant at 50 %. There were at least 15 air exchanges per hour and forced air heating was used to maintain temperature. For each generation, treatments were being applied until sample collection at 12 to 13 weeks old.

Feed and water were fed *ad libitum*. A starter game bird crumble (Purina Game Bird Startena, Purina Animal Nutrition, Arden Hills, MN) was fed from 0 to 6-7 weeks of age. A laying hen pellet (Purina Layena Pellets, Purina Animal Nutrition, Arden Hills, MN) was fed to both males and females from 6 to 7 to 17 weeks of age.

### Sample collection

Adult birds (16.86 weeks-old) were euthanized using cervical dislocation and brain, liver, kidneys, and thighs were collected. Tissues were placed into Whirlpak bags and immediately submerged into liquid nitrogen. Samples were then transferred to a −80°C freezer until further analyses.

After purchase of Purina® Gamebird Maintenance Feed (Purina Animal Nutrition, LLC, Arden Hills, MN 55126) 8 samples of feed were stored at −20°C. These samples were divided and analyzed immediately or subjected to either 22°C or 31°C for 48 hours to approximate the amount of time the feed was at those respective temperatures before consumption. The feed was analyzed at 48 hours of temperature exposure because feed was replaced every 48 hours; therefore, it was not necessary to analyze feed beyond this time point.

### Lipid Extraction

For adult brain, liver, and kidneys, 8 samples per treatment per sex were analyzed for fatty acid composition. For thighs, sample size varied (HS = 15, HSS = 16, TN = 12, TNS = 11).

Lipids were extracted using a modified Folch method (FOLCH et al., 1957; Zhang et al., 2017). For lipid extraction, samples were weighed according to their respective total % lipid to obtain 2 mg of total lipids. C19:0 triacylglycerol (Nu Chek Prep Inc, Elysian, MN; Cat# T-165) was added as an internal standard for thigh and liver samples and C19:0 phosphatidylcholine (Avanti Polar Lipids, Alabaster, Alabama; Cat #850367P) was added as an internal standard for brain and kidney samples because of its rarity in biological samples (Tokuşoğlu, 2006; De Marchi et al., 2011; Gecgel et al., 2015). Samples were then homogenized in 1 mL of methanol (for HPLC, ≥99.9 %, Sigma-Aldrich, St. Louis, MO, USA; Cat #34860; BioSpec, Tissue Tearor 398). The total contents of the homogenate were transferred to an 8 mL glass screw top test tube and 2 mL of chloroform and 0.75 mL of deionized water were added to each test tube. The mixture was vortexed and centrifuged at 458 × *g* for 10 minutes (Allegra 6 centrifuge with GH–3.8A rotor, Beckman Coulter, 128 Palo Alto CA). The bottom chloroform layer was transferred to a second test tube. Another 2 mL of chloroform were added to the first tube, vortexed, centrifuged, and the chloroform layer was again transferred to the previously mentioned second test tube. The chloroform containing the total extracted lipid was evaporated under N_2_ and reconstituted in 400 µl of toluene (ACS reagent, ≥99.5 %, Sigma-Aldrich, St. Louis, MO, USA; Cat #179418).

### Fatty acid composition

For fatty acid transesterification, 3 mL of methanol were added to the sample containing extracted lipids and toluene, followed by the addition of 600 µl of 8 % HCl (ACS reagent, 37 %, Sigma-Aldrich, St. Louis, MO, USA; Cat #320331) in methanol. Samples were vortexed and heated at 90°C on a dry heating block for 60 minutes and cooled to room temperature (∼23°C). Then, 1 mL each of hexane (for HPLC, ≥97.0 %, Sigma-Aldrich, St. Louis, MO, USA; Cat #34859) and deionized water were added before vortexing. Phase separation was allowed to occur for approximately 15 minutes and 900 µL of the top hexane layer were transferred to micro-centrifuge tubes containing 450 µl of deionized water. Micro-centrifuge tubes were centrifuged at 16,627 × g for 2 minutes at 4°C and the hexane layer was transferred to another set of tubes; solvents were evaporated under N_2_ and the remaining content reconstituted with 100 µL hexane (Zhang et al., 2017).

Reconstituted samples were transferred to amber gas chromatography vials containing inserts. Fatty acid methyl esters were analyzed on a Perkin-Elmer Clarus 500 gas chromatography system coupled to a flame ionization detector (PerkinElmer, Inc., Shelton, Connecticut, US). A DB-FFAP nitroterephthalic-acid-modified polyethylene glycol (PEG) capillary column (FFAP; 30 m × 0.25 mm inner diameter, 0.25 µm film thickness; Agilent Technologies, Santa Clara, California, US) was used. Trans and cis isomers were not disseminated because a non-specific flame ionizing detector was used. Initial oven temperature of 80°C was held for 2 minutes, then increased by 10°C/minute to 185°C at the time of sample injection. Then, the temperature increased by 5°C/minute to 240°C and was held at 240°C for 13 minutes. The injector and detector temperatures were 240°C and 300°C, respectively. Helium was the carrier gas at a flow rate of 1.3 mL/minute. The injection volume was 1 µL and the split ratio was set to 10:1. TotalChrom software (version 6.3.2.0646; PerkinElmer, Inc., Shelton, Connecticut, US) software was used for data collection and peak area integration. Peaks were identified based on their retention times, determined through injection of a standard commercial mixture of fatty acid methyl esters (Nu Chek Prep Inc, Elysian, MN). Concentration of fatty acids were calculated using the equation below.

$$\frac{\left(\frac{\text{Amount}\;\text{of}\;\left(C19:0\right)\left(\text{mg}\right)}{\text{Area}\;\text{of}\;\left(C19:0\right)}\right)\times \;\text{Area}\;\text{of}\;\text{FA}\;\text{peak}}{\text{Weight}\;\text{of}\;\text{sample}\;\left(g\right)}=\text{Concentration}\;\text{of}\;\text{FA}\;\left(\frac{\text{mg}}{g}\right)$$

The concentrations were then added together and individually divided by the total to obtain % of total FA as the final unit for statistical comparison.

### Statistical analyses

Analyses of data were performed in R 4.0.0 (R Core Team, 2020; RStudio Team, 2022) to determine significance (P ≤ 0.05). Differences in fatty acid composition were first examined using *t*-distributed stochastic neighbor embedding (t-SNE) (Van der Maaten and Hinton, 2008). This technique projected the concentration of all n = 29 of fatty acids in all n = 241 samples on a two-dimensional plot. We constructed the plot using the Rtsne package of R and visualized it with the ggplot2 package (Wickham, 2016). The value of perplexity was 40. Observations (samples) were colored by organ and treatment (HS, HSS, TN, TNS). Differences among treatments were determined using an ANOVA for the main effects and their interactions. The main effect for the adult tissues was treatment. Analysis of the feed was reported as an average with standard deviation. Appropriate post-hoc analysis was used if the interaction was significant at P ≤ 0.05. If an effect had significant differences, pairwise contrasts were made with confidence levels of 0.95 using Tukey’s method for comparing estimates.

## Results

### t-SNE of adult tissues and treatment

**Fig. 2:**
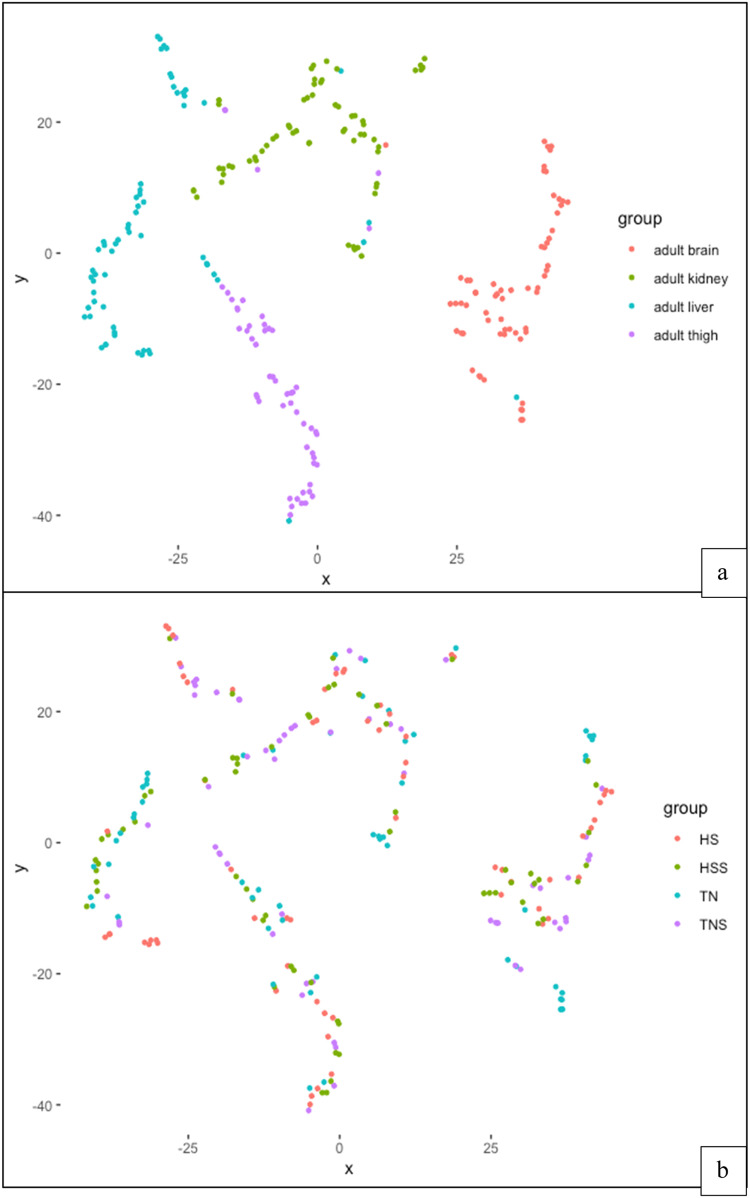
a-b. Adult fatty acids t-SNE (perplexity 40). (a) fatty acids colored by tissue type and (b) fatty acids colored by treatment.

With t-SNE, we projected the concentration of all fatty acids in samples in a two-dimensional plot. The samples clustered by tissue (brain, kidney, liver, thigh). There was little clustering by treatment (Figures 2a-b). This analysis showed that tissue was an important variable determining the fatty acid composition of samples. This was substantiated by the differences (P < 0.0001) of tissue when treatment was not a factor included in the analyses (Table 1).

**Table 1:** Fatty acid (%) composition^1^ compared by tissue.

Fatty Acids of Tissues (%)						
	Brain	Liver	Kidney	Thigh	P value	SD
C14:0^2^	0.21^a^	0.39^b^	0.11^c^	0.18^d^	<0.0001	0.17
C14:1^2^	0.076^a^	0.082^a^	0.019^b^	0.031^c^	<0.0001	0.051
C15:0^2^	2.32^a^	0.030^b^	0.60^c^	0.026^b^	<0.0001	0.96
C16:0^2^	23.07^a^	26.47^a^	7.27^b^	4.97^c^	<0.0001	13.97
C16:1^2^	0.62^a^	2.84^b^	0.91^ac^	1.11^c^	<0.0001	1.31
C17:0^2^	5.34^a^	0.11^b^	0.085^b^	0.041^c^	<0.0001	2.40
C18:0^2^	19.64^a^	0.095^b^	0.58^c^	2.85^d^	<0.0001	8.65
C18:1 cis/trans	16.00^a^	19.10^b^	16.20^a^	28.00^c^	<0.0001	7.28
C18:2 n-6^2^	0.78^a^	9.83^b^	9.15^b^	7.41^c^	<0.0001	4.98
C18:3 n-3^2^	0.31^a^	0.23^b^	0.27^ab^	0.29^ab^	<0.0001	0.16
C20:0^2^	0.23^a^	0.084^b^	0.10^c^	0.047^d^	<0.0001	0.11
C20:1 n-9^2^	0.27^a^	0.15^b^	0.11^b^	0.088^c^	<0.0001	0.11
C20:2 n-6	0.41^a^	0.17^b^	0.44^a^	0.041^c^	<0.0001	0.20
C20:3 n-6	0.27^a^	0.24^a^	0.37^b^	0.042^c^	<0.0001	0.16
C20:4 n-6^2^	9.82^a^	4.37^b^	5.03^c^	1.23^d^	<0.0001	3.30
C20:5 n-3^2^	0.33^a^	0.14^b^	0.057^c^	0.039^c^	<0.0001	0.15
C22:0	0.33^a^	0.091^b^	0.16^c^	0.040^d^	<0.0001	0.11
C22:1^2^	0.35^a^	0.25^b^	0.086^c^	0.071^d^	<0.0001	0.16
C22:2	3.13^a^	0.07^b^	0.21^c^	0.07^b^	<0.0001	1.27
C22:5 n-3^2^	0.45^a^	0.12^b^	0.11^b^	0.077^c^	<0.0001	0.16
C22:5 n-6^2^	5.00^a^	0.45^b^	0.18^c^	0.12^d^	<0.0001	2.19
C22:6^2^	12.89^a^	0.012^b^	0.18^c^	0.0029^d^	<0.0001	5.67
∑SFA^2, 4^	50.00^a^	57.40^b^	40.20^c^	29.50^d^	<0.0001	11.80
∑MUFA^4^	17.20^a^	22.60^b^	18.60^a^	32.30^c^	<0.0001	7.22
∑PUFA^4^	32.30^a^	23.10^b^	40.70^c^	36.80^d^	<0.0001	8.04
∑n-6 PUFA^4^	18.40^a^	20.60^b^	37.60^c^	33.60^d^	<0.0001	9.25
∑n-3 PUFA^4^	13.57^a^	2.48^b^	3.13^c^	2.92^bc^	<0.0001	4.91
∑SFA:PUFA^3^^,^^4^	1.56^a^	2.70^b^	0.99^c^	0.82^d^	<0.0001	0.94
∑n-6:n-3^4^	1.36^a^	9.10^b^	12.17^c^	11.81^c^	<0.0001	4.88

a-d Superscripts indicate significant differences at *P* ≤ 0.05 in rows.

1 Fatty acids were compared by tissue as the main effect.

2 Transformation of log (x) was used in the analysis but presented means and SD are presented in their original scale.

3 Transformation of 1/(x) was used in the analysis but presented means and SD are presented in their original scale.

4 SFA, saturated fatty acids; MUFA, monounsaturated fatty acids; PUFA, polyunsaturated fatty acids; n-6, omega-6 PUFA; n-3, omega-3 PUFA.

### Feed

ANOVA results showed that there were no temperature effects on any of the fatty acids, total SFA, MUFA, PUFA, n-6 PUFA, n-3 PUFA, SFA:PUFA, and n-6:n-3 (Table 2).

**Table 2:** Fatty acid (%) composition^1^ of stored feed^2^.

Fatty Acids of Feed (%)					
	−20°C^2^^,^^3^	22°C^2^^,^^3^	31°C^2^^,^^3^	P value	SD
C14:0	0.33	0.34	0.34	0.53	0.02
C14:1	nd^4^	nd^4^	nd^4^	nd^4^	nd^4^
C15:0	0.08	0.08	0.08	0.83	0.01
C16:0	2.50	2.53	2.52	0.19	0.46
C16:1	0.49	0.51	0.53	0.72	0.08
C17:0	0.16	0.17	0.16	0.67	0.01
C18:0	10.80	11.80	13.50	0.08	2.15
C18:1 cis/trans	16.50	15.20	14.90	0.20	1.78
C18:2 n-6	43.80	43.70	44.50	0.30	1.03
C18:3 n-3	11.40	11.30	12.00	0.59	1.37
C20:0	0.29	0.28	0.29	0.43	0.01
C20:1	0.58	0.60	0.60	0.45	0.03
C20:2 n-6	0.17	0.18	0.18	0.37	0.01
C20:3 n-6	0.01	0.02	0.02	0.15	0.01
C20:4 n-6	0.20	0.18	0.20	0.33	0.03
C20:5 n-3	0.06	0.05	0.06	0.37	0.02
C22:0	0.21	0.21	0.21	0.76	0.01
C22:1	0.06	0.06	0.07	0.48	0.01
C22:2 n-3	0.13	0.13	0.13	0.57	0.01
C22:5 n-3	nd^4^	nd^4^	nd^4^	nd^4^	nd^4^
C22:5 n-6	0.05	0.04	0.04	0.37	0.01
C22:6 n-3	0.21	0.21	0.21	0.69	0.01
∑SFA^5^	26.40	28.5	24.0	0.28	4.99
∑MUFA^5^	17.60	16.20	15.90	0.16	1.82
∑PUFA^5^	56.20	55.80	57.20	0.18	1.43
∑n-6 PUFA^5^	44.40	44.20	45.00	0.33	1.03
∑n-3 PUFA^5^	11.60	11.50	12.20	0.58	1.32
∑SFA:PUFA^5^	0.47	0.50	0.42	0.32	0.09
∑n-6:n-3^5^	3.87	3.83	3.74	0.87	0.43

1 Fatty acids were compared by temperature as the main effect.

2 Four treatments were: (1) thermoneutral controls (22°C, TN), (2) thermoneutral siblings (22°C, TNS), (3) heat stress (31°C, HS), and (4) heat stressed siblings (31°C, HSS) TN and HS were obtained through generational mating at 22°C and 31°C, respectively. TNS and HSS were obtained by mating males and females from TNS and dividing their offspring evenly into chambers at 22°C (TNS) and 31°C (HSS). Only families from TNS that had high fitness in HSS were mated.

3 20°C, feed storage temperature; 22°C, feed kept at temperature of thermoneutral and thermoneutral siblings’ treatments for 48 hours; 31°C, feed kept at temperature of heat stress and heat stress siblings’ treatments for 48 hours.

4 nd, not detected.

5 SFA, saturated fatty acids; MUFA, monounsaturated fatty acids; PUFA, polyunsaturated fatty acids; n-6, omega-6 PUFA; n-3, omega-3 PUFA.

### Adult brains

TN a higher ratio of SFA:PUFA and a lower ratio of n-6:n-3 fatty acids (P < 0.01). HS had more C14:0 and total MUFA than HSS (P = 0.01 and P < 0.001, respectively); however, there were no other significant differences between HS and HSS. Overall, most of the differences were between TN and all other treatments (P < 0.05; Table 3).

**Table 3:** Fatty acid (%) composition^1^ of adult brains compared by treatment^2^.

Fatty Acids of Adult Brains (%)						
Treatment						
	TN	TNS	HS	HSS	P value	SD
C14:0	0.25^a^	0.22^ab^	0.16^b^	0.23^a^	0.01	0.08
C14:1	0.11^a^	0.07^b^	0.06^b^	0.06^b^	<0.0001	0.03
C15:0	1.85^a^	2.18^ab^	2.42^bc^	2.49^c^	<0.0001	0.36
C16:0	23.40	23.30	22.60	23.00	0.49	1.63
C16:1	0.80^a^	0.63^ab^	0.54^b^	0.53^b^	<0.0001	0.24
C17:0	6.82^a^	4.55^b^	5.53^ab^	5.11^b^	<0.0001	1.46
C18:0	19.60	19.90	19.20	19.80	0.66	1.61
C18:1 cis/trans	18.90^a^	14.90^b^	15.90^b^	14.80^b^	<0.001	2.98
C18:2 n-6	0.74	0.74	0.74	0.69	0.91	0.32
C18:3 n-3	nd^3^	nd^3^	nd^3^	nd^3^	nd^3^	nd^3^
C20:0	0.32^a^	0.17^b^	0.25^ab^	0.20^ab^	0.03	0.14
C20:1	0.21	0.29	0.20	0.30	0.17	0.07
C20:2 n-6	0.52	0.35	0.44	0.36	0.06	0.20
C20:3 n-6	0.28	0.19	0.30	0.30	0.58	0.15
C20:4 n-6	9.66	10.06	9.80	9.88	0.62	0.94
C20:5 n-3	0.32	0.30	0.29	0.33	0.81	0.18
C22:0	0.43	0.38	0.34	0.33	0.42	0.13
C22:1	0.33	0.33	0.38	0.35	0.10	0.07
C22:2 n-3	2.99	3.21	3.21	3.00	0.28	0.36
C22:5 n-3	0.47	0.41	0.48	0.44	0.17	0.09
C22:5 n-6	4.49	5.02	4.98	5.49	0.13	1.17
C22:6 n-3	13.40^a^	13.30^ab^	12.20^b^	12.70^ab^	0.03	1.25
∑SFA^4^	48.30^a^	50.50^b^	50.30^b^	50.80^b^	<0.001	1.76
∑MUFA^4^	20.30^a^	16.00^b^	17.00^b^	15.90^b^	<0.001	3.17
∑PUFA^4^	29.80^a^	33.30^b^	32.20^b^	33.10^b^	<0.0001	1.94
∑n-6 PUFA^4^	16.10^a^	19.00^b^	19.00^b^	19.20^b^	<0.001	2.32
∑n-3 PUFA^4^	14.20	14.00	13.20	13.50	0.08	1.22
∑SFA:PUFA^4^	1.65^a^	1.53^b^	1.56^b^	1.54^b^	<0.01	0.08
∑n-6:n-3^4^	1.15^a^	1.37^b^	1.46^b^	1.43^b^	<0.01	0.23

a-c Superscripts indicate significant differences at *P* ≤ 0.05 in rows by treatment.

1 Fatty acids were compared by treatment as the main effect.

2 Four treatments were: (1) thermoneutral controls (22°C, TN), (2) thermoneutral siblings (22°C, TNS), (3) heat stress (31°C, HS), and (4) heat stressed siblings (31°C, HSS) TN and HS were obtained through generational mating at 22°C and 31°C, respectively. TNS and HSS were obtained by mating males and females from TNS and dividing their offspring evenly into chambers at 22°C (TNS) and 31°C (HSS). Only families from TNS that had high fitness in HSS were mated.

3 nd, not detected.

4 SFA, saturated fatty acids; MUFA, monounsaturated fatty acids; PUFA, polyunsaturated fatty acids; n-6, omega-6 PUFA; n-3, omega-3 PUFA.

### Adult kidneys

There were no differences between HS and HSS (P > 0.05). The most differences were seen between TN and TNS with there being more C18:0, less C20:1, more C20:5, less C22:0, less C22:2 than TNS (P < 0.05). TN also had less SFA than HSS (P = 0.04) and a lower n-6:n-3 than HS (P < 0.01; Table 4).

**Table 4:** Fatty acid (%) composition^1^ of adult kidneys compared by treatment^2^.

Fatty Acids of Adult Kidney (%)						
	Treatment					
Fatty acid	TN	TNS	HS	HSS	P value	SD
C14:0	0.25	0.24	0.26	0.27	0.59	0.06
C14:1	0.05	0.05	0.04	0.04	0.43	0.02
C15:0	1.43	1.54	1.51	1.45	0.79	0.33
C16:0	17.60	17.30	17.10	17.70	0.17	0.82
C16:1	2.43	1.83	1.79	2.01	0.12	0.79
C17:0	0.95	1.33	1.43	1.40	0.07	0.55
C18:0	19.90^a^	17.90^b^	19.60^a^	19.50^a^	<0.0001	1.67
C18:1 cis/trans	16.10	15.80	16.30	16.50	0.84	2.17
C18:2 n-6	21.00	23.60	21.40	22.00	0.09	3.06
C18:3 n-3	0.61	0.73	0.55	0.65	0.25	0.08
C20:0	0.23^ab^	0.27^b^	0.23^a^	0.23^a^	0.01	0.04
C20:1	0.24^a^	0.27^b^	0.26^ab^	0.27^b^	0.01	0.02
C20:2 n-6	1.00	1.11	1.20	1.12	0.34	0.29
C20:3 n-6	0.90	0.87	1.00	0.91	0.17	0.17
C20:4 n-6	12.90	12.50	13.00	11.80	0.42	2.23
C20:5 n-3	0.27^a^	0.11^b^	0.12^b^	0.12^b^	<0.0001	0.09
C22:0	0.37^a^	0.48^b^	0.38^a^	0.41^ab^	0.02	0.11
C22:1	0.39	0.01	0.20	0.19	<0.0001	0.07
C22:2	0.45^a^	0.56^b^	0.50^ab^	0.53^ab^	0.05	0.11
C22:5 n-3	0.28	0.28	0.26	0.28	0.60	0.06
C22:5 n-6	0.50	0.40	0.45	0.44	0.73	0.23
C22:6 n-3	2.26	2.19	1.98	2.02	0.20	0.43
∑SFA^3^	40.50^ab^	39.20^a^	40.30^ab^	41.00^b^	0.04	1.81
∑MUFA^3^	18.80	18.20	18.60	19.00	0.84	2.67
∑PUFA^3^	40.10	42.30	40.50	39.80	0.15	3.41
∑n-6 PUFA^3^	36.80	39.10	37.60	36.80	0.16	3.24
∑n-3 PUFA^3^	3.34^a^	3.29^a^	2.89^b^	3.02^ab^	<0.01	0.40
∑SFA:PUFA^3^	1.02^ab^	0.92^a^	0.99^ab^	1.04^b^	0.03	0.11
∑n-6:n-3^3^	11.10^a^	12.00^ab^	12.30^b^	12.30^ab^	<0.01	1.51

a-b Superscripts indicate significant differences at P 0.05 in rows by treatment.

1 Fatty acids were compared by treatment as the main effect.

2 Four treatments were: (1) thermoneutral controls (22°C, TN), (2) thermoneutral siblings (22°C, TNS), (3) heat stress (31°C, HS), and (4) heat stressed siblings (31°C, HSS) TN and HS were obtained through generational mating at 22°C and 31°C, respectively. TNS and HSS were obtained by mating males and females from TNS and dividing their offspring evenly into chambers at 22°C (TNS) and 31°C (HSS). Only families from TNS that had high fitness in HSS were mated.

3 SFA, saturated fatty acids; MUFA, monounsaturated fatty acids; PUFA, polyunsaturated fatty acids; n-6, omega-6 PUFA; n-3, omega-3 PUFA.

### Adult livers

TNS was the most different among the treatments with higher percentages of C18:2 n-6, C18:3 n-3, C22:2 n-3, total PUFA, and total n-6 PUFA and lower percentages of C14:0, C16:1, C18:1 cis/trans, and total MUFA (P < 0.0001). When considering pair differences, TNS and HSS had more differences than any other treatment pairs (P < 0.01; Table 5).

**Table 5:** Fatty acid (%) composition^1^ of adult livers compared by treatment^2^.

Fatty Acids of Adult Livers (%)						
	Treatment					
Fatty acid	TN	TNS	HS	HSS	P value	SD
C14:0	0.48^a^	0.28^b^	0.59^a^	0.60^a^	<0.0001	0.23
C14:1	0.09^ab^	0.03^a^	0.12^b^	0.10^b^	<0.0001	0.07
C15:0	0.03^a^	0.04^b^	0.03^a^	0.03^ab^	0.01	0.01
C16:0	25.80	25.10	27.60	27.80	0.08	3.48
C16:1	3.10^a^	1.91^b^	4.03^a^	3.61^a^	<0.0001	1.37
C17:0	0.12	0.10	0.11	0.13	0.42	0.04
C18:0	26.90^a^	37.00^b^	29.20^ab^	27.50^a^	0.01	13.98
C18:1 cis/trans	20.40^a^	10.90^b^	22.00^a^	22.70^a^	<0.0001	5.32
C18:2 n-6	13.20^a^	17.50^b^	10.00^a^	11.00^a^	<0.0001	3.86
C18:3 n-3	0.22^a^	0.35^b^	0.20^a^	0.20^a^	<0.0001	0.12
C20:0	0.08	0.06	0.06	0.07	0.45	0.04
C20:1	0.18	0.17	0.13	0.12	0.07	0.07
C20:2 n-6	0.25	0.19	0.19	0.16	0.15	0.11
C20:3 n-6	0.32	0.31	0.27	0.30	0.94	0.23
C20:4 n-6	6.39^ab^	6.87^b^	3.79^c^	4.25^ac^	<0.0001	2.80
C20:5 n-3	0.19	0.15	0.14	0.15	0.46	0.09
C22:0	0.13	0.12	0.08	0.08	0.04	0.06
C22:1	0.23^ab^	0.22^a^	0.24^ab^	0.26^b^	0.02	0.03
C22:2 n-3	0.11^a^	0.05^b^	0.11^a^	0.10^a^	<0.0001	0.06
C22:5 n-6	0.65	0.51	0.49	0.46	0.36	0.32
C22:5 n-3	0.15^ab^	0.18^b^	0.09^a^	0.12^ab^	0.01	0.07
C22:6 n-3	2.09^ab^	2.80^b^	1.12^a^	1.43^a^	<0.0001	1.26
∑SFA^3^	54.20	62.70	57.60	55.00	0.10	10.16
∑MUFA^3^	23.80^a^	12.90^b^	26.50^c^	26.40^ac^	<0.0001	6.15
∑PUFA^3^	24.10^a^	33.70^b^	17.60^c^	19.50^ac^	<0.0001	7.79
∑n-6 PUFA^3^	25.50^a^	28.10^b^	16.50^c^	17.50^ac^	<0.0001	6.45
∑n-3 PUFA^3^	2.61^ab^	3.44^b^	1.64^a^	2.04^a^	<0.01	1.40
∑SFA:PUFA^3^	2.36^ab^	1.71^a^	3.49^c^	2.99^bc^	<0.001	1.18
∑n-6:n-3^3^^,^^4^	9.40	7.77	9.90	9.15	0.23	2.94

a-c Superscripts indicate significant differences at *P* ≤ 0.05 in rows by treatment.

1 Fatty acids were compared by treatment as the main effect.

2 Four treatments were: (1) thermoneutral controls (22°C, TN), (2) thermoneutral siblings (22°C, TNS), (3) heat stress (31°C, HS), and (4) heat stressed siblings (31°C, HSS) TN and HS were obtained through generational mating at 22°C and 31°C, respectively. TNS and HSS were obtained by mating males and females from TNS and dividing their offspring evenly into chambers at 22°C (TNS) and 31°C (HSS). Only families from TNS that had high fitness in HSS were mated.

3 SFA, saturated fatty acids; MUFA, monounsaturated fatty acids; PUFA, polyunsaturated fatty acids; n-6, omega-6 PUFA; n-3, omega-3 PUFA.

4 Transformation of log (x) was used in the analysis but presented means and SD are presented in their original scale.

### Adult thighs

When considering all tissues, thighs had the least number of differences with 5 differences among treatments. Among these differences TN had lower C14:0, and higher C16:1 and total MUFA than HSS (P = 0.03; Table 6).

**Table 6:** Fatty acid (%) composition^1^ of adult thighs compared by treatment^2^.

Fatty Acids of Adult Thighs (%)						
	Treatment					
Fatty acid	TN	TNS	HS	HSS	P value	SD
C14:0	0.55^a^	0.62^ab^	0.67^b^	0.67^b^	0.03	0.12
C14:1	0.08	0.13	0.14	0.11	0.08	0.06
C15:0	0.08^a^	0.13^b^	0.09^a^	0.08^a^	0.002	0.02
C16:0	17.60	17.30	17.80	18.60	0.40	1.96
C16:1	4.49^a^	3.73^ab^	3.68^ab^	3.16^b^	0.03	1.17
C17:0	0.12	0.17	0.17	0.15	0.13	0.05
C18:0	10.30	10.40	11.10	10.80	0.76	2.06
C18:1 cis/trans	30.80	29.30	27.40	27.80	0.05	3.39
C18:2 n-6	26.70	29.10	27.80	27.40	0.39	3.42
C18:3 n-3	0.92	1.07	1.02	0.97	0.57	0.25
C20:0	0.16	0.17	0.18	0.16	0.52	0.05
C20:1	0.26	0.30	0.33	0.30	0.16	0.07
C20:2 n-6	0.14	0.13	0.16	0.16	0.68	0.04
C20:3 n-6	0.13	0.17	0.17	0.16	0.29	0.05
C20:4 n-6	5.13	4.86	5.15	5.02	0.99	2.07
C20:5 n-3	nd^3^	nd^3^	nd^3^	nd^3^	nd^3^	nd^3^
C22:0	0.16	0.19	0.19	0.14	0.47	0.07
C22:1	0.20^ab^	0.25^ab^	0.34^b^	0.22^a^	0.03	0.13
C22:2 n-3	0.26	0.25	0.33	0.31	0.51	0.13
C22:5 n-6	0.45	0.39	0.52	0.52	0.58	0.26
C22:5 n-3	0.27	0.29	0.31	0.32	0.75	0.12
C22:6 n-3	1.65	1.52	1.63	1.73	0.92	0.74
∑SFA^4^	29.00	28.70	29.40	30.50	0.61	3.68
∑MUFA^4^	36.10^a^	33.40^ab^	31.50^b^	31.60^b^	0.03	4.30
∑PUFA^4^	35.50	37.10	37.60	36.60	0.51	3.56
∑n-6 PUFA^4^	32.70	34.30	34.00	33.50	0.72	3.42
∑n-3 PUFA^4^	2.82	2.87	2.85	3.07	0.71	0.63
∑SFA:PUFA^4^	0.85	0.79	0.81	0.84	0.70	0.15
∑n-6:n-3^4^	11.90	11.80	12.30	11.30	0.67	2.18

a-b Superscripts indicate significant differences at *P* ≤ 0.05 in rows by treatment.

1 Fatty acids were compared by treatment as the main effect.

2 Four treatments were: (1) thermoneutral controls (22°C, TN), (2) thermoneutral siblings (22°C, TNS), (3) heat stress (31°C, HS), and (4) heat stressed siblings (31°C, HSS) TN and HS were obtained through generational mating at 22°C and 31°C, respectively. TNS and HSS were obtained by mating males and females from TNS and dividing their offspring evenly into chambers at 22°C (TNS) and 31°C (HSS). Only families from TNS that had high fitness in HSS were mated.

3 nd, not detected.

4 SFA, saturated fatty acids; MUFA, monounsaturated fatty acids; PUFA, polyunsaturated fatty acids; n-6, omega-6 PUFA; n-3, omega-3 PUFA.

## Discussion

### t-SNE

The strong association of fatty acids within tissue was expected because evidence shows that there are tissue-specific responses to changes in dietary fat (Marion and Woodroof, 1963; Surai and Sparks, 2000). This is because there are tissue-specific lipid and fatty acid metabolism; therefore, fatty acid composition varies by tissue function (Storch and Thumser, 2010).

The weak association between treatment and fatty acid was to be expected. Although heat stress has been shown to significantly alter the synthesis of unsaturated fatty acids in birds, the temperature chosen for this study may not have been high enough to illicit such responses (Yang et al., 2024). Antioxidant activity was not significantly different between treatment groups; therefore, there is evidence that selection for low FCR at 31°C does not result in a change in fatty acid profiles or an increase in protection of fatty acids through certain antioxidants like superoxide dismutase or catalase (Truong and King, 2023).

### Feed

Purina® Gamebird Maintenance Feed (Purina Animal Nutrition, LLC, Arden Hills, MN 55126) had a high level of stearic acid that contributed 10.8 % to 13.5 % of the total fatty acids in the feed. These values were higher than those in quail feed containing soybean oil (Szabó et al., 2010; Donaldson et al., 2017). The high level of stearic acid was closer to values when lard or animal fat is used in feed formulation (Donaldson et al., 2017). Animal fat has also been shown to have almost 3 times as much stearic acid than many plant-based oils (Bilska and Krzywdzińska-Bartkowiak, 2025). Although not listed as lard or animal fat, the porcine meat and bone meal, listed as the 3rd ingredient on the feed label, was most likely the origin of this high level of stearic acid. Otherwise, all fatty acids were in adequate amounts to support healthy birds (Alagawany et al., 2019).

In the current study, fatty acids were not affected by temperatures which demonstrate that the antioxidant feed additives, such as vitamin E, zinc oxide, and sodium selenite, were sufficient at preventing oxidation of the fatty acids, particularly dietary essential fatty acids such as linoleic acid and α-linolenic acid. Feed exposure to 31°C for 48 hours could also have been too short of a duration for significant amount of lipid oxidation to occur (Liu et al., 2019).

### Adult brain

The diet did not have many differences, therefore differences seen in tissues can be attributed to physiological changes due to selective breeding. DHA is 50-70 % of the fatty acids in brain synaptosome membranes which contributes to membrane fluidity, signal transduction, protein signaling, and gene expression (Cherian, 2005). Therefore, high abundance of DHA in the brain was to be expected. When DHA is not in adequate quantities, DPA takes its place in the brain (Catala, 2012). Contrary to the findings in this current study, others found that Japanese quail subjected to 42°C did not have significant lipid changes in the brain (Durairaj and Martin, 1971).

As expected, HS had significantly lower levels of DHA in the brain than all other treatments because this treatment group was not chosen to have high fitness in heat stress; therefore, more oxidation of DHA may have occurred prior to transport to the brain. The brain does not have desaturase activity, all the DHA present in the brain was transported via plasma from either the diet or after synthesis in the liver (Catala, 2012). Long chain PUFA are particularly susceptible to oxidation when an animal is experiencing heat stress (Noble and Cocchi, 1990). The results from the present study showed that TN brains had lower total PUFA than TNS and HSS, which was opposite to expectations. The higher level of total PUFA in TNS, HS, and HSS could indicate that quail in these treatments adapted to heat stress by increasing antioxidant response to protect compounds, including lipids, from oxidation. However, overall, previous data showed that quail brain had the most oxidation when compared to liver, kidney, and thigh and did not have significantly different catalase and superoxide dismutase activity when compared by treatment (Truong and King, 2023). Therefore, other factors must be considered such as heat shock proteins or desaturase activity that resulted in the higher level of total PUFA in TNS, HS, and HSS brains (Archana et al., 2017). A possible explanation could be that TNS, HS, and HSS experienced more neuroplasticity because has been associated with more PUFAs, particularly n-3, being incorporated into cell membranes of the brain (Reemst et al., 2022). The significantly higher ratio of SFA to PUFA and lower ratio of n-6 to n-3 in TN could indicate that there is more neuroplasticity in heat stressed animals or animals that were selected for high fitness in heat stress, but never experienced heat stress. It also appears that TNS, HS, and HSS brains had similar changes compared to TN, indicating that their brain function was altered by either selection for low FCR or heat stress.

### Adult Kidneys

Kidneys play an important role in regulating homeostasis and changes in their fatty acid profile could indicate stress in the animal (Alabdallah et al., 2021). There is evidence that heat stress causes renal damage and increases fatty acid transfer to the kidney because of an increase in urinary liver fatty acid binding protein which transfers long chain fatty acids to the kidney (Goto et al., 2024). The fatty acid transfer to the kidney is important for ATP production through beta oxidation (Goto et al., 2024). However, others have found an inhibition of other molecules directly involved in transportation of fatty acids to mitochondria and beta oxidation in the kidney (Wang et al., 2024). There was no treatment effect in DHA or ARA which are the long chain PUFA that are important for membrane fluidity to ensure proper sodium pump function (Szabó et al., 2010). In the present work, there were higher levels of stearic acid and total SFA in HSS than TNS indicating that quail that were chosen to have low FCR in heat stress and then exposed to heat stress likely had more renal stress than those that were chosen to have low FCR in heat stress but not later exposed to heat stress. Additionally, the lower levels of stearic acid and SFA in TNS may indicate less oxidative damage and cellular degradation than other treatments (Szczuko et al., 2020). The renal stress likely influenced the higher n-6:n-3 in HS than TN. This observation was also supported by the lower level of n-3 in HS than TN and TNS.

### Adult Livers

The liver is the source of elongation and desaturation of fatty acids in avian species and increased environmental temperatures result in an increase in oxidative stress in the liver (Cherian, 2011; Hosseini-Vashan and Raei-Moghadam, 2019; Tang et al., 2022). There is also preferential deposition of n-3 fatty acids over n-6 fatty acids from the liver to the yolk (Noble and Cocchi, 1990; Speake et al., 1998). This maternal control over fatty acids deposited into the yolk can influence the inflammation response in chicks because lower n-6:n-3 have been observed in decreased inflammation (Cherian, 2011). There is evidence of genetically linked responses to stressors; therefore, future studies should investigate if that selection for low FCR in poultry over time can result in an upregulation of protective n-3 PUFAs in preparation for a stressor as seen in the significantly higher levels of n-3 PUFA in TNS compared to HS and HSS (Soliva-Estruch et al., 2023). The higher level of LA in HS and HSS indicated a possible suppression of the conversion of ALA to EPA or DHA; therefore, a lower amount of n-3 was found in quail from the HS and HSS groups (Alagawany et al., 2019). From this study, birds that are chosen for high fitness in heat stress may be experiencing fatty acid changes in the liver when housed in thermoneutral temperatures but when exposed to heat stress, there is no fatty acid differences than those that were not chosen for low FCR.

### Thighs

The recommended n-6:n-3 is 4 to 1; however, the current western human diet has a ratio of 10:1 to 20:1 (Patterson et al., 2012; Dal Bosco et al., 2022). The current study had similar findings with adult quail thighs having an average n-6:n:3 of 11.8:1 (Table 6). Other researchers showed that tissue fat of fasting birds have an increase in SFA concentrations due to the preferential metabolism of unsaturated fatty acids (Ben-Hamo et al., 2013). Researchers also note that shorter chain unsaturated fatty acids are selectively mobilized for energy but in membranes, n-6 fatty acids such as ARA and LA are preferentially retained and may increase in concentration during fasting (Ben-Hamo et al., 2013). Birds tend to decrease feed intake during heat stress to decrease their heat increment from metabolizing feed (Naga Raja Kumari and Narendra Nath, 2018). However, there was no clear treatment effect indicating that HS and HSS had significantly less shorter chain unsaturated fatty acids, such as LA and ALA, than TN or TNS. This experiment showed that selection for low FCR at 31°C did not incur many fatty acid differences in the thigh which means this selection criteria for poultry may not offer a more beneficial fatty acid profile for human consumption.

Overall, this experiment showed that organs react differently to heat stress with the liver exhibiting more differences in fatty acids than brain, kidney, or thigh. Heat stress or selection for low FCR in heat stress may also result in more neuroplasticity due to higher levels of PUFA in the brain and altered brain function. Lastly, selection for low FCR in heat stress does not result in fatty acid changes in brain, liver, kidney, or thigh compared to those that are randomly bred in heat stress because across all tissues and fatty acids, there were only 3 differences between HS and HSS.

Future studies should include higher temperatures from 30°C and above to determine how fatty acid composition of quail and other commercial poultry are affected when subjected to selective breeding to withstand increasing temperatures during climate change. As well, it is important to continue the work of others to know if heat stress affects cognitive ability through the oxidation of fatty acids using Japanese quail as a model (Laviola and Macrì, 2013; Tallima and El Ridi, 2017). Future studies should also investigate the temperature at which changes in fatty acid composition in poultry occur and if selectively breeding for low FCR in heat stress results in genetic or epigenetic changes in subsequent generations.

## CRediT authorship contribution statement

**Linda Truong:** Conceptualization, Data curation, Formal analysis, Investigation, Methodology, Project administration, Validation, Visualization, Writing – original draft, Writing – review & editing. **Timothy J. Hackmann:** Writing – review & editing, Visualization, Formal analysis. **Zhichao Zhang:** Data curation, Methodology, Software. **Annie J. King:** Writing – review & editing, Writing – original draft, Supervision, Project administration, Methodology, Investigation, Funding acquisition, Conceptualization.

## Disclosures

The authors declare that they have no known competing financial interests or personal relationships that could have appeared to influence the work reported in this paper.
